# Boosting Photocatalytic Overall Water Splitting Activity of Phosphorene Through Five‐Coordinate Passivation Enabled by Carbene Addition

**DOI:** 10.1002/anie.9859942

**Published:** 2026-07-03

**Authors:** He Zhang, Yanbo Li, Junchi Xu, Shengkun Liu, Taotao Wang, Chao Gao, Pingwu Du, Hengxing Ji, Jun Jiang, Guan‐Wu Wang, Yujie Xiong, Jong‐Beom Baek, Shangfeng Yang

**Affiliations:** ^1^ State Key Laboratory of Precision and Intelligent Chemistry Collaborative Innovation Center of Chemistry for Energy Materials (*i*ChEM) School of Chemistry and Materials Science University of Science and Technology of China Hefei China; ^2^ Department of Energy and Chemical Engineering Center For Dimension‐Controllable Organic Frameworks Ulsan National Institute of Science and Technology Ulsan South Korea; ^3^ Hefei National Research Center for Physical Sciences at the Microscale Collaborative Innovation Center of Chemistry for Energy Materials (iChEM), School of Chemistry and Materials Science University of Science and Technology of China Hefei China; ^4^ Key Laboratory of Functional Molecular Solids Ministry of Education Anhui Laboratory of Molecule‐Based Materials, School of Chemistry and Materials Science Anhui Normal University Wuhu China

**Keywords:** adamantane, five‐coordinate passivation, phosphorene, phosphorus–carbon double bonds, photocatalytic overall water splitting

## Abstract

Phosphorene is a promising two‐dimensional semiconductor for solar‐driven redox reactions, yet its practical deployment is severely restricted by rapid degradation under ambient conditions. Conventional covalent functionalization typically forms phosphorus–carbon single bonds (P─C), leaving phosphorus atoms in a four‐coordinate environment and thus failing to fully quench the intrinsic reactivity associated with one residual unpaired electron. Here, we develop a selective strategy to achieve five‐coordinate passivation of phosphorene by constructing phosphorus–carbon double bonds (P═C) through a one‐step photochemical carbene addition reaction. Using a carbene precursor, adamantane groups are grafted onto phosphorene to afford a robust P═C‐bonded architecture. Comprehensive spectroscopic analyses, together with density functional theory (DFT) calculations, validate the preferential formation of the P═C bonds. The resulting P═C‐passivated phosphorene exhibits markedly improved ambient stability compared to the pristine and four‐coordinate‐passivated phosphorene. When utilized as a metal‐free photocatalyst, the P═C‐passivated phosphorene enables highly efficient overall water splitting without sacrificial agents under visible light, delivering record‐high evolution of H_2_ and H_2_O_2_ with rates of up to 612 and 658 µmol h^−1^ g^−1^, respectively, along with excellent cycling stability.

## Graphical abstract

We report selective five‐coordinate passivation of phosphorene via P═C double bonds using a facile one‐step photochemical reaction via carbene addition. The ambient stability of functionalized phosphorene has been substantially improved compared to the pristine phosphorene. Furthermore, the as‐formed adamantane grafted phosphorene exhibits remarkable photocatalytic overall water splitting activity for H_2_ and H_2_O_2_ coproduction.

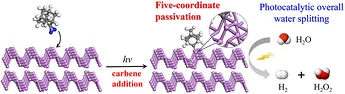

## 1 Introduction

Phosphorene as an emerging two‐dimensional (2D) nanomaterial exhibits thickness‐dependent direct bandgap and high charge carrier mobility, facilitating its prospective applications in catalysis and energy [1, 2, 3, 4]. However, owing to the reactive lone pair electrons on each phosphorus atom, phosphorene suffers from poor ambient stability and readily reacts with oxygen and moisture upon exposure in ambient conditions, severely hampering its applications [5]. With the destruction of the phosphorene structure, its unique properties such as high charge carrier mobility significantly decay, resulting in decreased performance [6, 7]. For instance, when the phosphorene‐based field effect transistor was exposed to humid oxygen for 19.5 h, the on‐current decreased by approximately two orders of magnitude [6]. Therefore, it is crucial to improve the ambient stability of phosphorene.

Among the reported methods for chemically passivating the vulnerable phosphorene, covalent functionalization is one of the most effective strategies, enabling the construction of covalent bonds between phosphorus atoms and the grafted group [8, 9, 10]. Since the seminal work of constructing phosphorus–carbon (P─C) single bonds via aryl diazonium chemistry by Hersam et al. in 2016, covalently passivating phosphorene via phosphorus–carbon (P─C) single bonds has been widely adopted through grafting small molecules such as azodiisobutyronitrile, alkyl halides, fullerene, resulting in significant improvement of the ambient stability of phosphorene [8, 9, 11, 12]. Nonetheless, upon forming P─C single bonds on phosphorus atom, a four‐coordinate mode phosphorus atom bearing three inherent P─P single bonds plus one P─C single bond is established [8]. This results in one residual unpaired electron remaining on the phosphorus atom and consequently inadequate passivation.

Alternatively, five‐coordinate passivation of phosphorene enables the additional bonding of this residual unpaired electron on the phosphorus atom; thereby more efficient passivation can be achieved, as demonstrated by the azide covalent passivation of phosphorene affording the formation of phosphorus‐nitrogen (P═N) double bonds reported by our group in 2019 [10]. Nevertheless, up to now, five‐coordinate passivation of phosphorene is predominantly limited to azide covalent passivation [10, 13, 14], whereas an analogous strategy constructing phosphorus–carbon (P═C) double bonds has remained elusive. To tackle this challenge, the successful synthesis of Wittig reagents containing P═C double bonds could guide the preparation of the P═C five‐coordinate phosphorene, as both involve formal phosphorus(V) centers [15, 16]. In particular, Wittig reagents could be directly synthesized from carbene addition to phosphine, in which a barrierless pathway exists when the vacant 2p_z_(C) atomic orbital of carbene is aligned with the phosphorus lone pair [17, 18].

In this study, we report the selective five‐coordinate passivation of phosphorene via P═C double bonds, achieved by a facile one‐step photochemical reaction of phosphorene with 2‐adamantane‐2,3‐[3*H*]‐diazirine (AdN_2_) via carbene addition. Few‐layer black phosphorus nanosheets (BPNSs), obtained from the electrochemical exfoliation of bulk black phosphorus (BP), react with the adamantane carbene intermediate generated from AdN_2_ under light irradiation at room temperature, affording stable adamantane‐grafted BPNSs (abbreviated as Ad‐BP) bearing P═C double bonds. A series of characterizations were carried out to verify the nature of five‐coordinate P═C double bonds in Ad‐BP. Density functional theory (DFT) calculations rationalize the selective formation of P═C double bonds, and a plausible reaction mechanism is also proposed. The as‐formed Ad‐BP featuring P═C double bonds exhibits drastically enhanced ambient stability relative to four‐coordinate‐passivated BPNSs. Moreover, Ad‐BP is utilized in photocatalytic overall water splitting for hydrogen (H_2_) and hydrogen peroxide (H_2_O_2_) coproduction, leading to evolution rates of 612 and 658 µmol h^−1^ g^−1^, respectively, without any sacrificial reagents. This performance outperforms all metal‐free photocatalysts and even most metal photocatalysts. As the first report on selective five‐coordinate passivation of phosphorene via P═C double bonds with superior photocatalytic overall water splitting performance, our work opens up a new avenue for covalent passivation of phosphorene toward energy conversion applications.

## 2 Results and Discussion

### 2.1 Synthesis and Characterization of Ad‐BP With P═C Double Bonds

**SCHEME 1:**
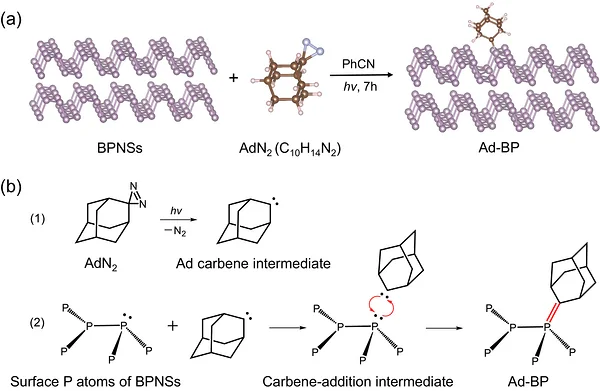
Synthesis route. (a) Preparation process and structure of Ad‐BP. (b) Proposed mechanism for the reaction between BPNSs and AdN_2_.

According to the previous reports in phosphorus chemistry, P═C double bonds could be kinetically stabilized by introducing bulky groups to shield the reactive center of phosphorus atom, and adamantane (Ad) has proven to be an ideal candidate for such a bulky groups [19]. Thus we covalently grafted Ad group onto BPNSs in our present work. Ad‐grafted BPNSs (abbreviated as Ad‐BP) were prepared via a one‐step photochemical reaction between phosphorene and AdN_2_, as illustrated in Scheme 1a.

Few‐layer BPNSs were prepared by electrochemical exfoliation of bulk BP as reported previously (Figure S1) [20]. AdN_2_ was synthesized according to the procedure reported in literature [21]. BPNSs and AdN_2_ were dispersed in benzonitrile (PhCN) in a Schlenk tube and degassed through a freeze‐pump‐thaw process, followed by light irradiation using a high‐pressure mercury‐arc lamp with a cut‐off filter (λ ≥ 350 nm) at room temperature for 7 h. Soluble byproducts were removed by washing with PhCN, tetrahydrofuran (THF), and ethanol repeatedly, yielding a black powder after drying in a vacuum.

The schematic reaction routes are shown in Scheme 1b. Generally, AdN_2_ is a typical precursor of a carbene (R_2_C:) intermediate [21]. Upon light irradiation, AdN_2_ first loses the [N═N] moiety to form a highly reactive carbene intermediate with two unpaired electrons, which subsequently reacts with BPNSs bearing lone pair electrons on phosphorus atoms, resulting in the formation of P═C double bonds via carbene addition reaction.

**FIGURE 1:**
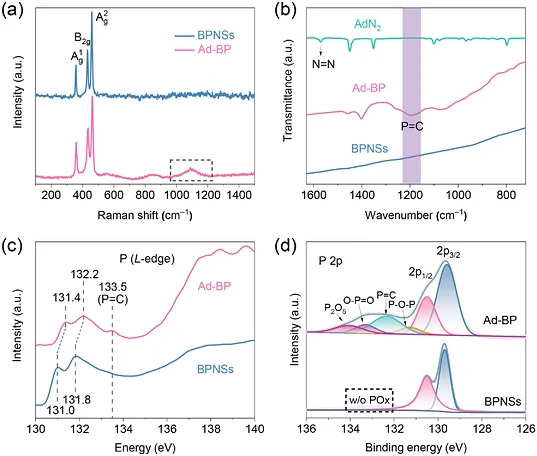
Spectroscopic characterizations. (a) Raman spectra of BPNSs and Ad‐BP. (b) FTIR spectra of AdN_2_, BPNSs, and Ad‐BP. (c) P L‐edge XAS spectra of BPNSs and Ad‐BP. (d) High‐resolution P2p XPS spectra of BPNSs and Ad‐BP.

A series of spectroscopic characterizations were carried out to unravel the bridging mode between the Ad group and BPNSs. The Raman spectrum of the pristine BPNSs shows three typical peaks of the $A_{g}^{1}$, $B_{2g}$, and $A_{g}^{2}$ vibration modes at 359, 434, and 462 cm^−1^, respectively (Figure 1a) [10]. After grafting Ad, a new broad signal centered at 1100 cm^−1^ appears in the Raman spectrum of Ad‐BP, which may be tentatively assigned to the newly formed covalent bonds between phosphorus (P) and carbon (C) atoms.

The characteristic peak for P─C single bonds at 700 cm^−1^ is absent [22], and thus the presence of P─C single bonds can be excluded. In addition, the relative Raman intensities of $A_{g}^{1}/A_{g}^{2}$ peaks of BPNSs and Ad‐BP are calculated as 0.38 and 0.45, respectively, indicating that carbene functionalization changes the chemical environment of the surface of BPNSs [10].

We then employed Fourier‐transform infrared (FTIR) spectroscopy so as to unveil the nature of the newly formed covalent bonds between P and C atoms (Figure 1b). For AdN_2_, the characteristic vibrational peaks at 1449, 1352, 1101, and 798 cm–^1^ are attributed to alkyl groups of adamantane, while the peak at 1572 cm^−1^ can be assigned to the diazirine (N═N) signal [23, 24]. BPNSs exhibit no discernible vibrational signals in the FTIR spectra. In contrast, the characteristic vibrational modes at 1459 and 1402 cm^−1^ in Ad‐BP are attributed to the alkyl groups of Ad and exhibit blue shifts in Ad‐BP, indicating the successful grafting of Ad group onto BPNSs. The characteristic vibrational peaks at 1260 and 1065 cm^−1^ are assigned to P═O and P─O groups, respectively, which arise because of the inevitable surface oxidation of phosphorene during the functionalization process [25].

Meanwhile, the N═N (1572 cm–^1^) vibrational mode is not observed in Ad‐BP, revealing the removal of the N═N group in the photochemical reaction along with the generation of carbene intermediate (Scheme 1b). Notably, a new vibrational signal appears at ∼1200 cm^−1^, which can be assigned to P═C double bonds [26]. This result is consistent with the reported P═C double bonds (∼1200 cm^−1^) in the functionalized phosphorene [26]. In addition, no obvious signal attributed to P─C single bonds (∼864 cm–^1^, Ref. [27]) is detected, demonstrating the selective P═C five‐coordinate passivation of phosphorene. In a previous report by Zhang et al. dichlorocarbene intermediate generated in situ from a chloroform (CHCl_3_) precursor under basic conditions reacted with BPNSs by using triethylbenzylammonium chloride as a phase transfer catalyst. This yielded a product mixture containing both P─C single bonds and P═C double bonds with inferior selectivity [26]. This highlights the advantage of our present method, which utilizes the photochemical generation of adamantane carbene intermediate in selective construction of P═C double bonds for the passivation of phosphorene.

To confirm the formation of P═C double bonds within Ad‐BP, we next performed x‐ray absorption spectroscopic (XAS) characterizations. The P L‐edge XAS spectra of BPNSs and Ad‐BP are compared in Figure 1c. The near‐edge absorption peaks in the region of 130–132.5 eV ascribed to the P (2p) → 1e* transition observed for the pristine BPNSs positively shift by ∼0.4 eV in the spectrum of Ad‐BP, suggesting the existence of charge transfer from BP to Ad [28, 29]. Furthermore, a new peak centered at ∼133.5 eV emerges in the spectrum of Ad‐BP, which is distinctly different from that of the P─C single bonds reported for covalently functionalized BPNSs (135.5 eV) [30], further excluding the existence of P─C single bonds. Given that there is no report of the XAS signal of P═C double bonds, we carried out simulations of the P L‐edge XAS spectra of BPNSs and Ad‐BP bearing P═C double bonds. The simulated XAS spectra of BPNSs and Ad‐BP agree well with the experimental results, especially regarding the predicted signal of P═C double bonds (∼133.6 eV, Figure S2). Moreover, Figure S3 compares the P K‐edge XAS spectra of BPNSs and Ad‐BP, which correspond to the electronic transitions from the P 1s core levels into the unoccupied P 3p levels and thus can be used to infer the interactions between BP and the grafted groups [31]. The absorption edge of Ad‐BP shifts positively by ∼ 0.5 eV relative to that of BPNSs, indicating electron transfer from BP to the grafted Ad groups, which is consistent with the result of the P L‐edge spectra [29]. In addition, Ad‐BP shows a new broad signal centered at ∼2168.0 eV, which might stem from the distortion of the P site induced by the bonded C atoms [29, 32]. We also performed XAS measurements on the photolyzed BPNSs (p‐BPNSs) and found that no obvious changes occurred in both P L‐edge and K‐edge XAS spectra before and after light treatment, excluding the illumination effect (Figure S4).

According to the x‐ray photoemission spectroscopic (XPS) survey spectra of BPNSs, there is no Pt element in BPNSs, indicating no Pt contamination during electrochemical exfoliation of bulk BP for preparing BPNSs (Figure S5) [20]. As depicted in the high‐resolution P 2p XPS spectra illustrated in Figure 1d, the pristine BPNSs show the characteristic P 2p_3/2_ and P 2p_1/2_ doublet peaks at 129.7 and 130.5 eV, respectively [9]. The sub‐band of oxidized phosphorus is negligible, indicating the preservation of the structural integrity of BPNSs during the electrochemical exfoliation process [20]. After grafting the Ad group, a distinct new peak at 132.4 appears, which can be attributed to P═C double bonds [33]. The other signals at 131.3, 133.3, and 134.1 eV are assigned to oxidized phosphorus species, including P─O─P (bridging bonding), O─P═O (dangling bonding), and P_2_O_5_ generated from the inevitable oxidation during the functionalization of BPNSs, which has been commonly reported in the literature related to the functionalized phosphorene materials [34, 35, 36, 37, 38]. Elemental analyses were performed to estimate the degree of functionalization of Ad‐BP. As shown in Table S1, the weight percentage of the C element is 5.97%, and the corresponding molar percentage of the Ad moiety is calculated to be 1.65%, indicating an average functionalization degree of ∼17 Ad moieties per 1000 phosphorus atoms in Ad‐BP.

The formation of P═C double bonds within Ad‐BP was further ascertained by employing solid‐state magic angle spinning (MAS) nuclear magnetic resonance (NMR) spectroscopy. Figure S6a shows the solid‐state ^13^C NMR spectra of Ad (C_10_H_16_) and Ad‐BP. Compared to Ad, Ad‐BP not only exhibits the signals of the Ad component but also a new broad peak centered at ∼129.3 ppm corresponding to P═C double bonds [39].

We also carried out simulations of the ^13^C NMR spectrum of Ad‐BP bearing P═C double bonds, and compared them with the experimental ^13^C NMR data. As shown in Figure S6b, the simulated solid‐state ^13^C NMR spectrum of Ad‐BP agrees well with the experimental one, especially regarding the calculated chemical shift of the carbon atom involved in the P═C double bond around 130 ppm.

On the other hand, ^31^P NMR spectra of BPNSs and Ad‐BP are also compared (Figure S6c). This comparison reveals that the single strong peak associated with the BP lattice at 20.8 ppm detected in BPNSs shifts upfield (negatively) to 19.2 ppm in Ad‐BP due to the increase of the bandgap as discussed below, resulting in increased nuclear shielding [10, 13]. Moreover, a new shoulder peak appears at −26.2 ppm in Ad‐BP, which may be associated with the P═C double bonds.

**FIGURE 2:**
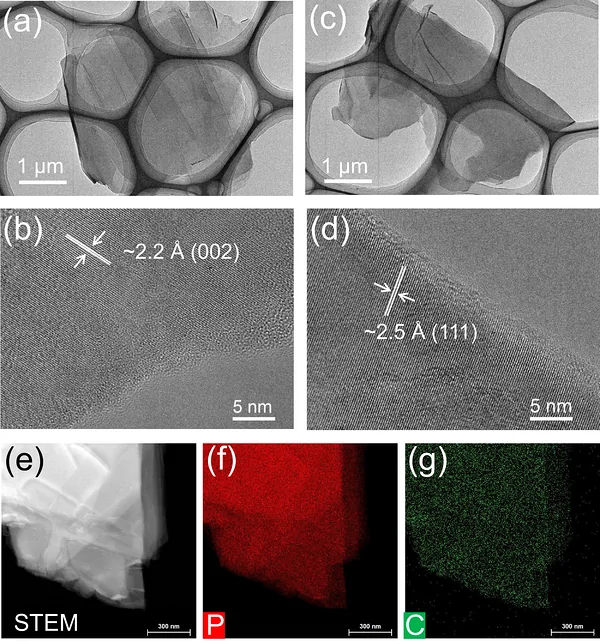
Microstructural characterizations. (a,c) Low‐magnification TEM images of BPNSs (a) and Ad‐BP (c, scale bar: 1 µm). (b,d) HRTEM images of BPNSs (b) and Ad‐BP (d, scale bar: 5 nm). (e–g) STEM and EDX elemental (P and C) mapping images of Ad‐BP (scale bar: 300 nm).

We next investigated the effects of Ad grafting on the morphology and crystallinity of BPNSs. Transmission electron microscopy (TEM) image of the pristine BPNSs shows individual platelets with a lateral size of about 4 µm, and the high‐resolution TEM (HRTEM) image reveals a (002) crystal plane spacing of about 2.2 Å (Figure 2a,b).

After Ad grafting, the TEM image of Ad‐BP shows that the nanosheets maintain a large size, indicating that the photochemical reaction imposes negligible influence on the nanosheet size and the crystallinity of BPNSs (Figure 2c). This result is consistent with the scanning electron microscopic (SEM) analyses (Figure S7). The HRTEM image of Ad‐BP exhibits lattice fringes of (111) plane of BP with a space of 2.5 Å (Figure 2d), suggesting Ad grafting does not destroy the crystallinity of BPNSs. This result is solidified by x‐ray diffraction (XRD) analyses (Figure S8), indicating the retention of the characteristic diffraction signals of BPNSs without obvious shifts.

Scanning transmission electron microscopy electron diffraction (STEM‐EDX) elemental mapping images of Ad‐BP show that the P and C elements are distributed over the entire nanosheets homogeneously, indicating the successful grafting of the Ad group on the surface of BPNSs (Figure 2e–g). Atomic force microscopic (AFM) images reveal comparable average thicknesses of BPNSs (∼3.76 nm) and Ad‐BP (∼3.69 nm), demonstrating that Ad grafting does not significantly affect the thickness of BPNSs (Figure S9).

### 2.2 DFT Calculations on the Formation Mechanism of Ad‐BP Bearing P═C Double Bonds

**FIGURE 3:**
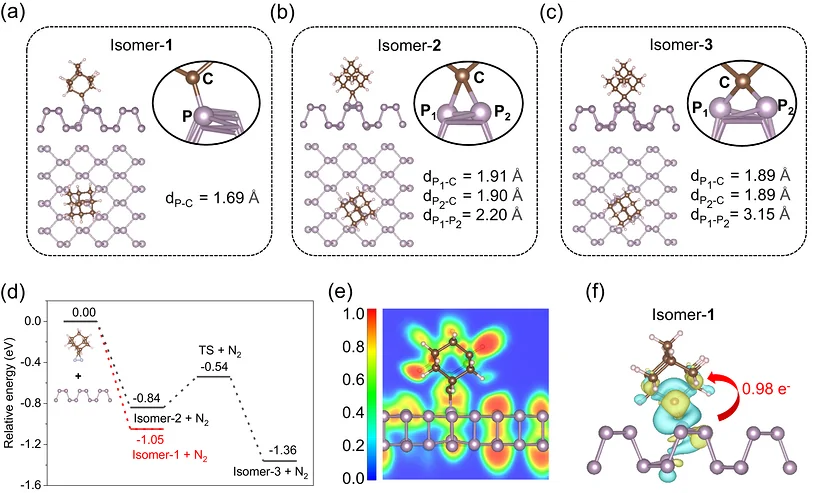
DFT calculations. (a–c) Optimized structures of three Ad‐BP isomers. The inserted figure shows a magnified view of the bonding regions near the P═C/P─C bonds. (d) Energy profiles for the formation of the adsorption between BPNSs and AdN_2_. The sum of the energies of BPNSs and AdN_2_ is used as the respective energy reference. (e) Color‐filled ELF maps of Isomer‐**1**. (f) Charge density difference of Isomer‐**1**. The yellow and blue isosurfaces correspond respectively to the charge accumulation and depletion regions after Ad adsorption.

To rationalize the selective formation of the P═C double bonds within Ad‐BP, we performed DFT calculations to investigate the reaction pathways of BPNSs and AdN_2_. DFT calculations indicate that the covalent bonding between BPNSs and Ad can lead to three possible products, with their structures shown in Figure 3a–c. Isomer‐**1** features a P═C double bond connecting the Ad group to a single phosphorus atom on the BP surface, with the optimized bond length of the P═C double bond being 1.69 Å (Figure 3a). Isomer‐**2** consists of an Ad bonded to two adjacent phosphorus atoms on the BP surface in a zigzag arrangement via two P─C single bonds. The bond lengths of the P─C bonds are 1.91 and 1.90 Å, and the P─P distance between the two phosphorus atoms is 2.20 Å (Figure 3b). Isomer‐**3** exhibits a similar bonding configuration to isomer‐**2**, with the Ad also connected to the BP surface via two P─C single bonds. However, the key difference lies in the P‐P distance, which increases significantly to 3.15 Å, while the bond lengths of the two P─C single bonds in Isomer‐**3** (1.89 Å) remain almost identical to those in Isomer‐**2** (Figure 3c). This rearrangement is driven by the elongation and opening of the constrained P–P bond in the strained P–C–P triangular‐ring motif of Isomer‐**2**, which effectively releases the local ring strain and consequently yields the more relaxed Isomer‐**3** configuration.

The formation pathways for these isomers are shown in Figure 3d. Isomer‐**1** is the most favorable product, forming through a barrierless process and exhibiting an adsorption energy of −1.05 eV. In contrast, Isomer‐**2**, also formed through a barrierless process, has a relatively lower adsorption energy of −0.84 eV, making it thermodynamically less stable than Isomer‐**1**. While Isomer‐**3** demonstrates a more favorable adsorption energy of –1.36 eV, its formation involves overcoming a barrier of 0.3 eV, making it kinetically less favorable. The formation mechanisms of P═C double bonds and P─C single bonds differ fundamentally: the barrierless formation of Isomer‐**1** involves the donation of the phosphorus lone pair electrons to carbon's vacant p‐orbital [18], whereas the alternative pathway requires P─P bond cleavage and significant structural reorganization, thereby introducing an energy barrier. Hence, based on the comprehensive thermodynamic and kinetic results, we conclude that Isomer‐**1** is the dominant product responsible for the selective formation of P═C double bonds instead of P─C single bonds.

The formation of the P═C double bonds in Isomer‐**1** was further confirmed by the electron localization function (ELF) analysis (Figure 3e). The ELF results reveal strong electron localization in the P═C bonding region, characterized by a two‐lobed distribution perpendicular to the bond axis, which indicates π‐electron localization and confirms the presence of the P═C double bonds [40, 41]. Notably, the high ELF values around the hydrogen and phosphorus atoms are attributed to the localized 1s orbital of hydrogen and the lone pair electrons on phosphorus. To determine the charge transfer between BPNSs and Ad, we performed differential charge density analysis (Figure 3f), which suggests charge depletion around P atoms and charge accumulation around C atoms. The Bader charge analysis shows that the number of electrons transferred from BPNSs to Ad is 0.98, which is higher than the values of 0.69 and 0.90 calculated for Isomer‐**2** and Isomer‐**3**, respectively (Figure S10). Such an intramolecular charge transfer is expected to affect the ambient stability and photocatalytic properties of BPNSs as discussed in detail below.

### 2.3 Effect of Ad Grafting on the Ambient Stability of BPNSs

To assess the effect of Ad grafting on the ambient stability of BPNSs, we carried out a UV–vis absorption spectroscopic study to monitor the optical absorbance of dispersions of Ad‐BP and BPNSs in water (without deoxygenation), which were stored under ambient conditions for one month. As shown in Figure S11a–c, the UV–vis absorbance of BPNSs at 460 nm degrades gradually, with the degradation ratio reaching 76% after standing for 30 days, indicating the poor ambient stability of BPNSs as reported previously [5]. After Ad grafting, Ad‐BP exhibits dramatic enhancement in ambient stability, as evidenced by the much smaller degradation ratio of 7% after standing for 30 days under the same conditions. This translates to ∼11‐fold enhancement of the ambient stability of BPNSs in terms of the inhibited degradation ratio for 30 days. Furthermore, to examine whether five‐coordinate passivation via P═C double bonds is superior to the four‐coordinate approach, a control four‐coordinate‐passivated BPNSs (4CP‐BPNSs) sample was prepared by covalently passivating BPNSs with an aryl diazonium (namely 4‐nitrobenzene‐diazonium) according to the procedure reported in the literature [8], and its stability was monitored in the same way. As shown in Figure S11d, the stability of Ad‐BP is about 18 times higher than that of 4CP‐BPNSs after standing for 13 days. Therefore, these results verify that five‐coordinate passivation via P═C double bonds is significantly superior for enhancing the ambient stability of BPNSs relative to four‐coordinate passivation via P─C single bonds, due to saturated coordination state of the phosphorus atom (Table S2).

To give more insight into the ambient stability of Ad‐BP, controlled‐environment stability tests were also conducted. Figure S12 shows that Ad‐BP exhibited comparable stabilities in an aprotic solvent (DMF) and water. Moreover, the stability of the P═C double bonds within Ad‐BP against water is validated by the characterization of water‐treated Ad‐BP (Figure S13), and theoretical calculations including ab initio molecular dynamics (AIMD) simulations and calculations the energy barrier for the reaction between Ad‐BP and H_2_O (Figures s14 and S15).

### 2.4 Photocatalytic Overall Water Splitting Performance of Ad‐Bp

Phosphorene‐based materials have been widely applied in the half‐reaction of photocatalytic hydrogen evolution using sacrificial agents, whereas studies on photocatalytic overall water splitting are rare because of the higher requirements for photocatalysts [42, 43]. Compared to the half‐reaction, overall water splitting eliminates the need for sacrificial agents, making it more applicable to large‐scale production [44, 45]. For the photocatalytic overall water splitting, the two‐electron process (2H_2_O → H_2_ + H_2_O_2_) is more kinetically favorable than the four‐electron process (2H_2_O → 2H_2_ + O_2_) [46]. In terms of the economic impact, the merits of the two‐electron process over four‐electron process include eliminating the cost of gas separation and generating H_2_O_2_, a much more valuable product than O_2_ [47, 48]. To the best of our knowledge, up to now, phosphorene‐based materials have never been reported for photocatalytic overall water splitting for H_2_ and H_2_O_2_ coproduction with quantitative detection of H_2_O_2_ [38]. The unprecedented five‐coordinate mode featuring P═C double bonds, along with the dramatic improvement in the ambient stability of Ad‐BP, motivated us to investigate the potential of Ad‐BP for photocatalytic overall water splitting for H_2_‐H_2_O_2_ coproduction.

**FIGURE 4:**
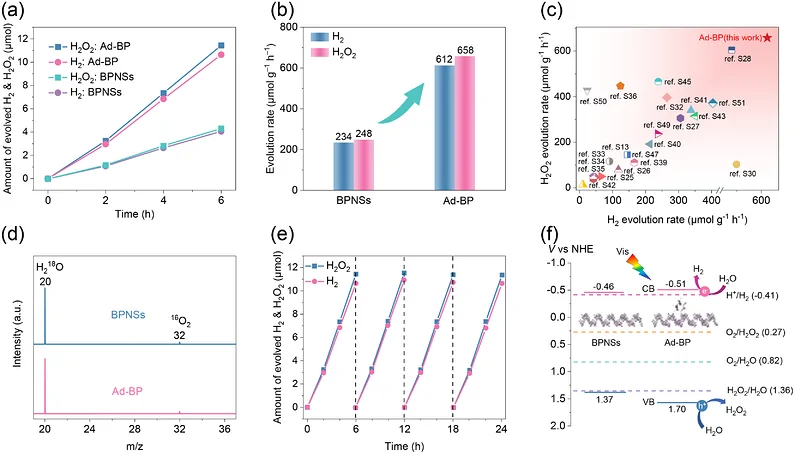
Photocatalytic overall water splitting into H_2_ and H_2_O_2_ over the BP‐based photocatalysts. (a) Dependence of H_2_O_2_ and H_2_ evolutions from water on time course under visible light irradiation (*λ* > 420 nm) by Ad‐BP and BPNSs as photocatalysts. (b) Comparison of the H_2_ and H_2_O_2_ evolution rates of BPNSs and Ad‐BP for photocatalytic pure water splitting. (c) Comparison of the photocatalytic performance in pure water splitting into H_2_ and H_2_O_2_ of recently reported photocatalysts. (d) SVUV‐PIMS spectrometry at photon energy of 13 eV of Ad‐BP and BPNSs of the gaseous products after 6 h photocatalysis under visible light. The reaction solution consisted of 1 mL H_2_^18^O, and 1 mg sample was used as the photocatalyst. (e) The cyclic stability of Ad‐BP for H_2_O_2_ and H_2_ evolutions under visible light. (f) Proposed schematic illustration showing the mechanism of the photocatalytic pure water splitting into H_2_ and H_2_O_2_ in the BPNSs and Ad‐BP.

The photocatalytic experiments were implemented in a batch reactor at room temperature by using a 300 W Xe lamp equipped with a UV cut‐off filter (*λ* > 420 nm). The amounts of H_2_ and H_2_O_2_ were quantified by gas chromatography (GC) and cerium sulfate (Ce(SO_4_)_2_) spectrophotometry, respectively [46]. For the latter case, the yield of H_2_O_2_ can be calculated from the calibration curve between the Ce^4+^ concentration and the intensity of the UV–vis absorption peak at 316 nm of Ce(SO_4_)_2_ following the method reported in the literature (Figure S16) [46]. As shown in Figure 4a, without any sacrificial reagents, both the pristine BPNSs and Ad‐BP afford H_2_ and H_2_O_2_ coproduction, and the H_2_ and H_2_O_2_ production rates reach 612 and 658 µmol g–^1^ h–^1^, respectively, for Ad‐BP, which are ∼2.6 times higher than those obtained from the pristine BPNSs (Figure 4b). Based on these standard mass‐normalized production rates, Ad‐BP delivers the highest H_2_ and H_2_O_2_ production rates among all reported metal‐free photocatalysts, outperforming most reported metal‐based photocatalysts evaluated under equivalent conditions without any sacrificial reagents (Figure 4c, see also Table S3).

Notably, the evolution rate ratio of H_2_ to H_2_O_2_ obtained for both BPNSs and Ad‐BP is close to 1:1, suggesting that water splitting into H_2_ and H_2_O_2_ catalyzed by BPNSs and Ad‐BP is a two‐electron process [46]. To ascertain the absence of oxygen (O_2_) in the photocatalytic product, we performed an H_2_^18^O isotopic experiment using Synchrotron vacuum ultraviolet photoionization mass spectrometry (SVUV‐PIMS) [46]. Figure 4d presents the gas products of photocatalytic overall water splitting of H_2_^18^O by BPNSs and Ad‐BP. For both BPNSs and Ad‐BP, peaks at *m*/*z* = 20 and 32 are assigned to the fragments of H_2_^18^O and ^16^O_2_, respectively, where the appearance of ^16^O_2_ originates from air contamination. No peak at *m*/*z* = 36 (^18^O_2_) is detected, indicating that no O_2_ generation occurs via the four‐electron process for BPNSs and Ad‐BP.

The mechanism of H_2_O_2_ production was further explored by electron paramagnetic resonance (EPR) measurements of hydroxyl radicals (·OH) using 5,5‐dimethyl‐1‐pyrroline *N*‐oxide (DMPO) as a spin‐trapping agent. As illustrated in Figure S17a,b, both BPNSs and Ad‐BP exhibit four distinct characteristic peaks for DMPO‐·OH adducts (1:2:2:1 quartet pattern), indicating the generation of ·OH radicals as intermediates to form H_2_O_2_ through H_2_O oxidation during the photocatalytic process [46]. These results confirm that water splitting photocatalyzed by BPNSs and Ad‐BP predominantly proceeds through the two‐electron pathway under Ar‐deaerated conditions (2H_2_O → H_2_ + H_2_O_2_).

We further performed photocatalytic experiments under different atmospheres (Ar, Air, and O_2_) to confirm the mechanism of H_2_ and H_2_O_2_ production over Ad‐BP. As shown in Figure S18, the H_2_ evolution rate decreases along with an increase in the H_2_O_2_ evolution rate with increasing O_2_ concentration, indicating that H_2_O_2_ could also be produced through O_2_ reduction. In contrast, the standard photocatalytic overall water splitting experiment exclusively proceeds through the two‐electron pathway under an Ar‐purged anaerobic atmosphere, effectively preventing any competing oxygen reduction pathways.

Stability is a critical parameter for a photocatalyst [30]. We next carried out a cycling stability test on Ad‐BP in pure water under an Ar atmosphere. Figure 4e shows the H_2_ and H_2_O_2_ evolution rates of Ad‐BP, which remain almost constant after four cycles, indicating that Ad‐BP has excellent cycling stability. In addition, negligible changes are observed in the XRD pattern and TEM image of Ad‐BP before and after the photocatalytic cycle stability test (Figures S19 and S20), indicating that the morphology and crystal structure of Ad‐BP are well preserved during the photocatalytic process. Such superior stability is consistent with the drastic enhancement of the ambient stability induced by five‐coordinate passivation via P═C double bonds, as discussed above.

To elucidate the origin of the enhanced photocatalytic activity of Ad‐BP relative to the pristine BPNSs, photocurrent response measurements and electrochemical impedance spectroscopy (EIS) were performed to investigate the corresponding charge separation dynamics. Ad‐BP delivers a much higher photocurrent response than the pristine BPNSs (Figure S21a), indicating that Ad‐BP has a stronger charge separation ability than the pristine BPNSs. According to a comparison of the EIS Nyquist plots of the pristine BPNSs and Ad‐BP, Ad‐BP exhibits a smaller arc radius (Figure S21b), suggesting that Ad‐BP has a faster charge‐transfer process than the pristine BPNSs. These results reveal that the higher photocatalytic performance of Ad‐BP for water splitting into H_2_ and H_2_O_2_ benefits mainly from the more efficient charge separation and transfer processes.

Finally, we measured the band structures of BPNSs and Ad‐BP using Mott–Schottky (MS) plots as well as UV–vis absorption spectra in order to elucidate the mechanism of the enhanced photocatalytic activity of Ad‐BP. For the pristine BPNSs and Ad‐BP, the measured conduction band (CB) and valence band (VB) positions are located at –0.46/1.37 V vs. NHE and –0.51/1.70 V vs. NHE, respectively (Figures S22 and S23; Table S4). This indicates that the CB shifts negatively while a more pronounced positive shift of the VB occurs for Ad‐BP relative to the pristine BPNSs. Accordingly, we plot an energy level diagram and propose a plausible mechanism as illustrated in Figure 4f. For BPNSs and Ad‐BP, the two‐electron process for H_2_O_2_ formation is kinetically more favorable and can be achieved at a higher reaction rate than the four‐electron process for O_2_ evolution, even if the VB energy level allows the formation of either O_2_ or H_2_O_2_ [38, 49]. Upon Ad grafting, crucially, Ad‐BP exhibits significantly enhanced chemical and structural stability compared with BPNSs, which effectively prolongs its long‐term operational durability during photocatalysis. Meanwhile, Ad‐BP shows a more negative CB and more positive VB, leading to more reductive photogenerated electrons and more oxidative holes compared to BPNSs. Simultaneously, the as‐formed P═C double bonds within Ad‐BP accelerate photogenerated carrier transfer and inhibit the charge carrier recombination, resulting in an enhanced photocatalytic overall water splitting activity.

## 3 Conclusion

In summary, the selective five‐coordinate passivation of phosphorene via P═C double bonds was achieved for the first time, through a facile one‐step photochemical reaction of phosphorene with AdN_2_ via carbene addition. The selective formation of P═C double bonds in the as‐formed Ad‐BP was confirmed by Raman, FTIR, XAS, XPS and solid‐state NMR spectroscopies, which excluded the existence of byproducts containing P─C single bonds. DFT calculations reveal that the formation of P═C double bonds is thermodynamically and kinetically favorable in Ad‐BP. Ad grafting leads to drastically enhanced ambient stability relative to both pristine BPNSs and four‐coordinate‐passivated BPNSs. Moreover, as the first report on photocatalytic overall water splitting for H_2_ and H_2_O_2_ coproduction with quantitative H_2_O_2_ detection, Ad‐BP delivered high H_2_ and H_2_O_2_ evolution rates of 612 and 658 µmol h^−1^ g^−1^, respectively, without any sacrificial reagents. Their rates represent the highest values among all reported metal‐free photocatalysts and even higher than those of most reported metal photocatalysts. The selective five‐coordinate functionalization of phosphorene via P═C double bonds developed in this work not only offers new insights into phosphorus chemistry but also opens up a new avenue for energy conversion applications of phosphorene.

## Author Contributions

**He Zhang**: writing – original draft, conceptualization, investigation, methodology, and formal analysis. **Yanbo Li**: validation, methodology, formal analysis and, investigation. **Junchi Xu**: investigation, validation, software, and data curation. **Shengkun Liu**: methodology, formal analysis, validation, and visualization. **Taotao Wang**: investigation, validation, and methodology. **Chao Gao**: investigation, validation, and methodology. **Pingwu Du**: methodology, software, and investigation. **Hengxing Ji**: methodology, validation, and software. **Jun Jiang**: visualization, supervision, data curation, methodology, and resources. **Guan‐Wu Wang**: resources, formal analysis, methodology, and data curation. **Yujie Xiong**: investigation, methodology, software, and resources. **Jong‐Beom Baek**: writing – review and editing, funding acquisition, supervision, resources, and conceptualization. **Shangfeng Yang**: writing – review and editing, writing – original draft, conceptualization, supervision, data curation, resources, and validation.

## Conflicts of Interest

The authors declare no conflicts of interest.

## Supporting information



The authors have cited additional references within the Supporting Information.
**Supporting File**: anie73439‐sup‐0001‐SuppMat.docx.

## Data Availability

The data that support the findings of this study are available from the corresponding author upon reasonable request.
