# Supplementary material for: Boosting Photocatalytic Overall Water Splitting Activity of Phosphorene Through Five‐Coordinate Passivation Enabled by Carbene Addition
Source: Angew Chem Int Ed Engl. 2026 Jul 3;65(36):e9859942. doi: 10.1002/anie.9859942 (PMC13528460; doi:10.1002/anie.9859942)
Supplement: Supplementary file 1 [file ANIE-65-e9859942-s001.docx]

Supporting Information

**Boosting Photocatalytic Overall Water Splitting Activity of Phosphorene through Five-Coordinate Passivation Enabled by Carbene Addition**

He Zhang^+^, Yanbo Li^+^, Junchi Xu, Shengkun Liu, Taotao Wang, Chao Gao, Pingwu Du, Hengxing Ji, Jun Jiang,^*^ Guan-Wu Wang,^*^ Yujie Xiong,^*^ Jong-Beom Baek,^*^ Shangfeng Yang^*^

Table of contents

[S1. Experimental Methods	3](#_Toc210502163)

[S2. Supporting Figures	6](#_Toc210502164)

[S3. Supporting Tables	29](#_Toc210502165)

[References	34](#_Toc210502166)

# S1. Experimental Methods

**Preparation of BPNSs.** The delamination process of bulk BP was carried out using a two-electrode system under an inert atmosphere. The applied voltage during the delamination process was −10 V. The cathode was BP crystal (30 mg) and the anode was platinum foil. The electrodes were set in parallel with a constant distance of 2.00 cm. A 0.002 M TBA·CH_3_COO/DMF (anhydrous and deoxygenated) was used as the electrolyte. After delamination, the dispersion of exfoliated BPNSs was quickly transferred to a centrifuge tube and centrifuged at 3000 rpm for 5 min to remove unexfoliated and thick-layered BP flakes. Next, the supernatant was decanted for further characterization and reaction. It was confirmed by the XPS survey spectra that the BPNSs have no Pt contamination during the delamination process consistent with previous literature.^[1]^

**Preparation of Ad-BP and p-BPNSs.** AdN_2_ was obtained from the procedure in the previous literature.^[2]^ Then, a PhCN solution of BPNSs and AdN_2_ in a Schlenk tube was degassed through a freeze-pump-thaw process. The reactor was gently stirred under an inert atmosphere and simultaneously irradiated using a high-pressure mercury lamp with a filter cutting off the light under 350 nm at room temperature for 7 h. After completion of the reaction, the product was separated from the mixture by centrifugation at 15000 rpm for 30 min, washed with PhCN, THF, and ethanol repeatedly, and then collected and dried under vacuum for 1 day. For comparison in Solid-state ^31^P NMR and XAS characterization, p-BPNSs as a control sample were prepared by similar procedures except without adding AdN_2_.

**DFT calculations.** All DFT calculations were performed using the Vienna Ab initio Simulation Package (VASP).^[3]^ The core electrons were described using projected augmented wave (PAW) potentials with a kinetic energy cutoff of 500 eV. We employed the Perdew, Burke, and Ernzerhof (GGA-PBE) generalized gradient approximation for exchange-correlation, including van der Waals corrections via the Grimme DFT-D3 method.^[4,5]^ A supercell containing 48 phosphorus atoms was used, with the convergence criteria for energy and force was set to 10^−5^ eV and 0.02 eV/Å. The structure optimization was carried out using a 3×2×1 k-point grid from the Monkhorst-Pack scheme for Brillouin zone sampling,^[6]^ and the calculation of ELF, CDD, and Bader charge analysis were performed with a 4×4×1 k-point grid. Transition states were calculated using a combination of the climbing image nudged elastic band (CI-NEB) method^[7]^ and the Dimer method.^[8]^ The adsorption energy of each covalent compound is defined as ΔE = E(Ad-BP) + E(N_2_) - E(BPNSs) - E(AdN_2_), where E(Ad-BP) is the energy of Ad-BP, E(N_2_) is the energy of the released nitrogen during the reaction process, E(BPNSs) and E(AdN_2_) are the initial energies of pristine BPNSs and AdN_2_, respectively. The simulation of XAS spectra was carried out using the FDMNES software,^[9]^ utilizing SCF calculations and Green's functions, with a cutoff radius set to 6 Å and the Efermi parameter set to -3. The NMR shielding tensors^[10]^ output by VASP were used without the G=0 contribution. The ab initio molecular dynamics (AIMD) simulation was performed on interfacial water systems by introducing 57 water molecules into the Ad-BP surface. The AIMD simulation was carried out in the canonical (NVT) ensemble using a Nose-Hoover thermostat at 300 K.^[11,12]^. The system was first equilibrated for 5 ps, followed by a 15 ps production run for data collection, with a time step of 1.0 fs.

**Characterizations.** Fourier-transform infrared (FTIR) spectra were performed on a TENSOR 27 spectrometer (Bruker, Germany) at room temperature. The Raman spectra were obtained at room temperature in Via Raman Microscope equipment (Renishaw, England) with a 532 nm excitation laser. Solid-state ^31^P and ^13^C nuclear magnetic resonance (NMR) spectra were carried out on AVANCE NEO 600 magic angle spinning (MAS) measurements (Bruker, Germany) using a standard Bruker 3.2 mm MAS probe with a spinning speed of 11 kHz. The high-resolution transmission electron microscopy (HR-TEM) and scanning transmission electron microscopy energy dispersive X-ray (STEM-EDX) mapping images were obtained on a JEOL-2100F (Rigaku, Japan) microscope operating at a voltage of 200 kV. The scanning electron microscopic (SEM) images were obtained from a JEOL JSM-6390LA instrument (Rigaku, Japan). X-ray photoelectron spectroscopy (XPS) was conducted on a Thermo-VV ESCALAB 250 (Thermo-VV Scientific) machine. Atomic force microscopy (AFM) images were obtained using a Dimension Icon SPM (Bruker, Germany). X-ray diffraction (XRD) patterns were obtained from a Smart Lab 9 kW X-ray diffraction instrument (Rigaku, Japan). UV–vis absorption spectroscopy was performed on a 3600 UV–vis spectrometer (Shimadzu, Japan). The phosphorus L-edge X-ray absorption spectroscopy (XAS) was collected with a 0.1 eV energy resolution at beamline 12B-α of the National Synchrotron Light Source (Hefei, China) at the National Synchrotron Radiation Laboratory. The P K-edge XAS measurements were conducted on beamline 4B7A of the Beijing Synchrotron Radiation Facility.

**Stability measurements.** The Ad-BP and BPNSs powders were dispersed in water and sonicated for 30 min. The Ad-BP and BPNSs aqueous solutions were kept under ambient condition for different periods, and the optical absorbance at each time point was monitored so as to investigate their stability according to the change of the optical absorbance intensity.

**Photocatalytic measurements.** Photocatalytic overall water splitting experiments were carried out on a photocatalytic system with a 200 mL Pyrex top-irradiation reaction vessel connected to a glass closed gas circulation system (Labsolar-III (AG), Beijing Perfectlight Technology Co. Ltd, China). A 300 W Xe-lamp equipped with a 420 nm cut-off filter was used to provide the visible light irradiation (λ > 420 nm). To select thin-layered samples to match the band gap for overall water splitting into H_2_ and H_2_O_2_, the dispersion of samples was centrifuged at 9000 rpm for 20 min to remove thick-layered BP flakes and the supernatant obtained was further centrifuged at 15000 rpm for 30 min to collect samples for photocatalytic measurements. In a typical photocatalytic reaction, 3 mg of the powder sample was dispersed in a 60 mL pure water. Before irradiation, the system was deaerated by Ar bubbling into the dispersion for 30 min and the dispersion was sonicated for 10 min. The amount of evolved H_2_ during irradiation was determined with a GC equipped with a thermal conductivity detector (TCD). The cerium sulfate (Ce(SO_4_)_2_) spectrophotometry approach was used to determine the H_2_O_2_ concentration.^[13]^ The yellow Ce^4+^ solution becomes a colorless Ce^3+^ solution after being reduced by H_2_O_2_. The concentration of Ce^4+^ was detected using a UV‒vis spectrophotometer with an obvious absorption peak at 316 nm. Therefore, the H_2_O_2_ concentration can be calculated from the amount of Ce^4+^ consumed during the reaction, as shown in equation (1). Typically, after the photocatalytic reaction, the suspension was centrifuged (15000 rpm) and then filtered with a 0.22 μm filter to remove the catalyst. The obtained solution was mixed with a yellow Ce^4+^ solution and used for further detection of H_2_O_2_.

2Ce^4+^ + H_2_O_2_ → 2Ce^3+^ + 2H^+^ + O_2_ (1)

**Photoelectrochemical measurements.** Photoelectrochemical measurements were performed at 25 ± 1 °C in a conventional three-electrode single-compartment quartz cell on an electrochemical station (CHI 760E). The illumination was provided by a 300 W Xe lamp equipped with a UV cutoff filter (λ > 420 nm). First, the as-obtained catalyst (1 mg) was dispersed in ethanol (1 mL) mixed with Nafion (100 μL), ultrasonicated for 30 min to form a homogenous ink. Then, the slurry was spin-coated onto an FTO glass electrode (3.0 × 1.0 cm) to form an active area (~1.0 cm^2^), further followed by vacuum drying. The counter electrode and reference electrode consisted of Pt foil and Ag/AgCl electrode, respectively. The photocurrent response, EIS, and MS were measured in 0.5 M Na_2_SO_4_ solution.

**Synchrotron vacuum ultraviolet photoionization mass spectrometry (SVUV-PIMS).** SVUV-PIMS was used to detect the oxygen production in the photocatalytic system at the mass spectrometry beamline (BL04B) of the National Synchrotron Radiation Laboratory at Hefei, China. The gaseous products were introduced into the ionization chamber through a deactivated fused-silica capillary (inner diameter 250 mm). Afterward, they were ionized using vacuum ultraviolet light and analyzed with a custom-built time-of-flight mass spectrometer.

**Phosphorus L-edge X-ray Absorption Spectroscopy (XAS) measurements.** The powder samples were uniformly mounted onto conductive sample holders and transferred into the soft X-ray analysis chamber under an ultra-high vacuum. Phosphorus L_2,3_-edge X-ray absorption spectroscopy (XAS) spectra were collected at beamline 12B-α of the Hefei Light Source, National Synchrotron Radiation Laboratory (NSRL), Hefei, China, with an energy resolution of 0.1 eV. The incident photon energy was scanned across the P L_2,3_-edge region, and the resulting sample signal was normalized to the incident photon flux. Multiple scans were collected for each sample to ensure spectral reproducibility, followed by standard background subtraction and normalization for comparison among different samples.

# S2. Supporting Figures

**Figure S1.** Photograph of electrochemical exfoliation equipment for the two-electrode system.

**Figure S2.** (a) Simulated P L-edge XAS spectra of BPNSs and Ad-BP. (b) Simulated and experimental P L-edge XAS spectrum of Ad-BP.

**Figure S3.** P K-edge XAS spectra of Ad-BP and BPNSs.

**Figure S4.** P L-edge and K-edge XAS spectra of BPNSs and p-BPNSs.

**Figure S5.** XPS survey spectra of electrochemically exfoliated BPNSs.

**Figure S6.** (a) Solid-state ^13^C NMR spectra of Ad and Ad-BP. (b) Simulated and experimental solid-state ^13^C NMR spectra of Ad-BP. (c) Solid-state ^31^P NMR spectra of Ad-BP, BPNSs and p-BPNSs.

**Figure S7.** SEM images of BPNSs (a) and Ad-BP (b).

**Figure S8.** XRD patterns of Ad-BP and BPNSs.

**Figure S9.** AFM images of BPNSs (a) and Ad-BP (b). Insets: corresponding height images of BPNSs and Ad-BP. The scale bars are 2 μm and 2 μm in (a) and (b), respectively.

**Figure S10.** Charge density difference of Isomer-**2** and Isomer-**3**. The yellow and blue isosurfaces correspond to the charge accumulation and depletion regions, respectively, after Ad adsorption.

**Figure S11.** UV/Vis absorption spectra of the pristine BPNSs (a) and Ad-BP (b) dispersed in water after exposure to air for 0, 2, 3, 6, 8, 11, 13, 16, 18, 21, 30 days. (c) Variation of the absorption ratios at 460 nm (A/A_0_) of the pristine BPNSs and Ad-BP after exposure to air for 0–30 days. (d) Degradation ratios (1-A/A_0_) of the pristine BPNSs, 4CP-BPNSs and Ad-BP after exposure to air for 0-13 days.

**Figure S12.** Variation of the absorption ratios at 460 nm (A/A_0_) of the pristine BPNSs and Ad-BP after exposure to air in water or DMF for 13 days.

**Figure S13.** P L-edge XAS spectrum (a) and Solid-state ^13^C NMR spectrum (b) of water-treated Ad-BP.

**Figure S14.** Evolution of energy and P=C bond length during the AIMD simulations. The inset represents the structure at the corresponding time. Orange circles mark the P=C double bond. The P=C bond length remains stable at approximately 1.7 Å throughout the AIMD trajectory, and no P–O bond formation is observed, indicating that Ad-BP is stable in water and would not undergo further oxidation to produce the phosphine oxide.

**Figure S15.** Free energy profile for (a) H_2_O dissociation, and (b) P-O formation reactions. The inserts show the collective variable (CV) used in the slow-growth calculation. When an H_2_O molecule approaches the P=C double bond, it first undergoes H_2_O dissociation with an energy barrier of 1.43 eV (a). Subsequently, as the OH group moves closer to the P atom, an additional barrier of 1.09 eV must be overcome to form the P-O bond (b). These two steps involve exceptionally high energy barriers, suggesting that the next step of hydrolysis can hardly happen. The reason responsible for the high energy barrier may be that Ad is a bulky group, and the effect of steric hindrance makes it difficult for H_2_O molecules to attack P=C double bonds within Ad-BP.

**Figure S16.** (a) UV‒vis absorption spectra of Ce^4+^ solutions with different concentrations. (b) The linear relationship between the absorbance and Ce^4+^ concentration.

**Figure S17.** The DMPO spin-trapping ESR spectra of (a) Ad-BP and (b) BPNSs in dark and after 15 s visible light irradiation.

**Figure S18.** Comparison of the H_2_ and H_2_O_2_ evolution rates of Ad-BP for photocatalytic pure water splitting in different atmospheres.

**Figure S19.** TEM image of Ad-BP after photocatalytic reaction under visible light irradiation for four cycles (24 h).

**Figure S20.** XRD pattern of Ad-BP before and after photocatalytic reaction under visible light irradiation for four cycles (24 h).

**Figure S21.** (a) Photocurrent responses. (b) Electrochemical impedance spectra over BPNSs and Ad-BP photocatalysts in 0.5 M Na_2_SO_4_ aqueous solution under visible light (λ > 420 nm) irradiation.

**Figure S22.** Mott-Schottky (MS) plots of the BPNSs and Ad-BP at various frequencies. E_CB_ vs NHE can be determined by the intercept on the x-axis in the MS plots.^[13,14]^

**Figure S23.** UV-vis absorption spectra of the pristine BPNSs (a) and the Ad-BP (c). (αhν)^2^ versus hν curve of the pristine BPNSs (b) and the Ad-BP (d). BP is a direct bandgap material, thus r=2 for Tauc plot (αhv)^r^ versus hν (ref. [15]).

# S3. Supporting Tables

**Table S1**. Weight contents of N, C, H elements within Ad-BP based on elemental analyses

| sample | Weight (mg) | N (%) | C (%) | H (%) |
| --- | --- | --- | --- | --- |
| Ad-BP | 1.322 | 0.03 | 5.97 | 0.87 |

Based on the weight percentage of C element is 5.97%, the molar ratio of Ad and P atoms can be calculated by:

$$n\left( \mathrm{Ad} \right):n\left( P \right)= \frac{\frac{5.97\%}{12\times10}}{\frac{1-\frac{5.97\%}{10\times12}\times134}{31}} = 1.65\%$$

Where 12 and 31 are the relative atomic weights of C and P atoms, respectively, and 134 is the molecular weight of the Ad moiety (C_10_H_14_).

**Table S2.** Comparison of our work with other five-coordinate passivation methods and the selected four-coordinate passivation methods (focusing on P-C and P-N single bonds) reported in recent years

| Passivation type | Bonding type | Reaction type | Reaction reagent | Reference |
| --- | --- | --- | --- | --- |
| Five-coordination passivation | P=C | Carbene reaction | 2-Adamantane-2,3-[3H]-diazirine | This work |
|  | P=C and P-C | Carbene reaction | Chloroform | 16 |
|  | P=N | Azide reaction | 4-Azidobenzoic acid | 17 |
|  |  |  | tosyl,  p-Methoxycarbonylphenyl,  4-trifluoroacetylphenyl, and adamantyl azides | 18 |
|  |  |  | Glycidyl azide polymer | 19 |
| Four-coordination passivation | P-C | Diazonium reaction | 4-Nitrobenzene-diazonium and  4-methoxybenzenediazonium tetrafluoroborate salts | 20 |
|  |  | Nucleophilic substitution | Nucleophilic reagents | 21 |
|  |  | Carbon-free radical reaction | Azodiisobutyronitrile | 22 |
|  |  | Solid-state mechanochemical reaction | Fullerene | 15 |
|  | P-N | Ultrasonic reaction | g-C_3_N_4_ | 23 |
|  |  | Thermal coupling reaction | g-C_3_N_4_ | 24 |

**Table S3.** Comparison of the H_2_ and H_2_O_2_ generation rates of Ad-BP with other reported photocatalysts for pure water splitting

| Catalysts | Solution | λ/nm | H_2_/μmol g^−1^ h^−1^ | H_2_O_2_/μmol g^−1^ h^−1^ | Reference |
| --- | --- | --- | --- | --- | --- |
| Ad-BP | H_2_O | > 420 | 612 | 658 | This work |
| graphene@Au | H_2_O | ≥ 420 | 65.6 | 49.7 | 25 |
| 2.8%-Cu/TiO_2_ | H_2_O | 250–400 | 118 | 80 | 26 |
| PCN-10-CP-10 | H_2_O | simulated solar light | 305.8 | 305.8 | 27 |
| CdS@ZnIn_2_S_4_ | H_2_O | *>* 400 | 540.3 | 604.8 | 28 |
| ZCS/PO/Ni_3_Pi_2_ | H_2_O | ≥ 420 | 1464 | 1375 | 29 |
| few-layer C_60_ nanosheets | H_2_O | ≥ 420 | 91 | 116 | 13 |
| Pt-KCN(5) | H_2_O | 400-800 | 550 | 103 | 30 |
| P-CoNi/ZIS | H_2_O | simulated solar light | 1228.7 | 1105.5 | 31 |
| DAnTMS/CD | H_2_O | 420-700 | 265.0 | 396.7 | 32 |
| C-N-g-C_3_N_4_ | H_2_O | 420- 700 | 42 | 49 | 33 |
| T-MCN-2 | H_2_O | *>* 420 | 48 | 47.76 | 34 |
| ISQDs-2.25 | H_2_O | / | 42.17 | 38.65 | 35 |
| PCN-Ni-CoP | H_2_O | simulated solar light | 124 | 447 | 36 |
| NiFe-P/ GDY | H_2_O | ≥ 420 | 928 | 674 | 37 |
| Bi_4_O_5_Br_2_ | H_2_O | / | 1149 | 447 | 38 |
| CoP/RP/BCN | H_2_O | > 420 | 166.2 | 110.4 | 39 |
| g-C_3_N_4_-Ni | H_2_O | > 400 | 213.2 | 191.9 | 40 |
| Pd_0.03_/ZIS | H_2_O | > 420 | 337.9 | 340.2 | 41 |
| Pt/C_3_N_4_ | H_2_O | *>* 400 | 10.80 | 13.80 | 42 |
| PCN@HP | H_2_O | > 420 | 350 | 315 | 43 |
| Pt/NaCN | H_2_O | 400-800 | 900 | 880 | 44 |
| CoP/CDs | H_2_O | ≥ 420 | 239 | 466 | 45 |
| Ni@grey anatase TiO_2_ | H_2_O | 325 | ≈700 | ≈700 | 46 |
| Ni_4_P_2_-CQDs@CdS | H_2_O | 420 | 145 | 145 | 47 |
| CoO-TS-1 | H_2_O | 420–700 | 1460 | 1390 | 48 |
| g-C_3_N_4_/AgIn*_x_*S*_y_* | H_2_O | > 420 | 237 | 237 | 49 |
| MOC-Q3/EA-COF | H_2_O | ≥ 420 | 23.51 | 428.21 | 50 |
| CoO_x_/ZIS | H_2_O | *>* 400 | 404.1 | 371.9 | 51 |
| BPD-COF | H_2_O | > 420 | 57 | 45 | 52 |
| Ni-CN-Co_2_P | H_2_O | > 420 | 91.5 | 85.5 | 53 |

**Table S4.** CB, VB energy levels and bandgaps of BPNSs and Ad-BP

| Sample | Bandgap (E_g_, eV) | Valence band (E_VB_, vs NHE) | Conduction band (E_CB_, vs NHE) |
| --- | --- | --- | --- |
| BPNSs | 1.83 | 1.37 | −0.46 |
| Ad-BP | 2.21 | 1.70 | −0.51 |

Bandgap (E_g_) is estimated from (αhν)^2^ versus hν curve shown in Supporting Information Figures S19b, d.
E_CB_ is determined by MS plots in Supporting Information Figures S18a, b.

E_VB_= E_g_ − |E_CB_|.

# References

[1] Wang, N. Mao, Z. Wang, X. Yang, X. Zhou, H. Liu, S. Qiao, X. Lei, J. Wang, H. Xu, X. Ling, Q. Zhang, Q. Feng, J. Kong, *Adv. Mater*. **2021**, *33*, e2005815.
[2] T. Sumanovac, M. Aleskovic, M. Sekutor, M. Matkovic, T. Baron, K. Mlinaric-Majerski, C. Bohne, N. Basaric, *Photochem. Photobiol. Sci.* **2019**, *18*, 1806–1822.
[3] G. Kresse, J. Furthmüller, *Comput. Mater. Sci.* **1996**, *6*, 15–50.
[4] J. P. Perdew, K. Burke, M. Ernzerhof, *Phys. Rev. Lett.* **1996**, *77*, 3865–3868.
[5] S. Grimme, S. Ehrlich, L. Goerigk, *J. Comput. Chem.* **2011**, *32*, 1456–1465.
[6] H. J. Monkhorst, J. D. Pack, *Phys. Rev. B* **1976**, *13*, 5188–5192.
[7] G. Henkelman, B. P. Uberuaga, H. Jónsson, *J. Chem. Phys.* **2000**, *113*, 9901–9904.
[8] G. Henkelman, H. Jónsson, *J. Chem. Phys.* **1999**, *111*, 7010–7022.
[9] O. Bunau, Y. Joly, *J. Phys.: Condens. Matter.* **2009**, *21*, 345501.
[10] J. Mason, *Solid State Nucl. Magn. Reson.* **1993**, 2, 285.
[11] S. Nosé, *The Journal of Chemical Physics* **1984**, 81, 511–519.
[12] W. G. Hoover, *Phys. Rev. A* **1985**, 31, 1695.
[13] T. Wang, L. Zhang, J. Wu, M. Chen, S. Yang, Y. Lu, P. Du, *Angew. Chem. Int. Ed.* **2023**, *62*, e202311352.
[14] T. N. Q. Trang, N. T. G. Bao, P. B. Thang, V. T. Hanh Thu, *Mater. Chem. Phys.* **2024**, *322*, 129568.
[15] X. Zhu, T. Zhang, D. Jiang, H. Duan, Z. Sun, M. Zhang, H. Jin, R. Guan, Y. Liu, M. Chen, H. Ji, P. Du, W. Yan, S. Wei, Y. Lu, S. Yang, *Nat. Commun.* **2018**, *9*, 4177.
[16] Y. Liu, P. Gao, T. Zhang, X. Zhu, M. Zhang, M. Chen, P. Du, G. W. Wang, H. Ji, J. Yang, S. Yang, *Angew. Chem. Int. Ed.* **2019**, *58*, 1479–1483.
[17] L. Zhang, Z.-J. Wang, B. Ma, X.-Y. Li, Y.-C. Dai, G. Hu, Y. Peng, Q. Wang, H.-L. Zhang, *Chinese Chem. Lett.* **2022**, *33*, 4640–4644.
[18] K. L. Walz Mitra, C. H. Chang, M. P. Hanrahan, J. Yang, D. Tofan, W. M. Holden, N. Govind, G. T. Seidler, A. J. Rossini, A. Velian, *Angew. Chem. Int. Ed.* **2021**, *60*, 9127–9134.
[19] S. Li, Y. Jiao, S. Ding, D. Yang, Z. Niu, G. Li, X. Wang, Y. Luo, *J. Mater. Sci.* **2022**, *57*, 17265–17276.
[20] C. R. Ryder, J. D. Wood, S. A. Wells, Y. Yang, D. Jariwala, T. J. Marks, G. C. Schatz, M. C. Hersam, *Nat. Chem.* **2016**, *8*, 597–602.
[21] Z. Sofer, J. Luxa, D. Bouša, D. Sedmidubský, P. Lazar, T. Hartman, H. Hardtdegen, M. Pumera, *Angew. Chem. Int. Ed.* **2017**, *56*, 9891–9896.
[22] H. Hu, H. Gao, L. Gao, F. Li, N. Xu, X. Long, Y. Hu, J. Jin, J. Ma, *Nanoscale* **2018**, *10*, 5834–5839.
[23] M. Zhu, S. Kim, L. Mao, M. Fujitsuka, J. Zhang, X. Wang, T. Majima, *J. Am. Chem. Soc.* **2017**, *139*, 13234.
[24] X. Wang, R. K. M. Raghupathy, C. J. Querebillo, Z. Liao, D. Li, K. Lin, M. Hantusch, Z. Sofer, B. Li, E. Zschech, I. M. Weidinger, T. D. Kuhne, H. Mirhosseini, M. Yu, X. Feng, *Adv. Mater.* **2021**, *33*, e2008752.
[25] J. Liang, Y. Liu, Z. Si, G. Wei, D. Weng, F. Kang, *Carbon* **2021**, *178*, 265–272.
[26] D. Wei, Y. Tan, Y. Wang, T. Kong, S. Shen, S. S. Mao, *Sci. Bull.* **2020**, *65*, 1389–1395.
[27] F. Xue, Y. Si, C. Cheng, W. Fu, X. Chen, S. Shen, L. Wang, M. Liu, *Nano Energ.* **2022**, *103*, 107799.
[28] E. Zhang, Q. Zhu, J. Huang, J. Liu, G. Tan, C. Sun, T. Li, S. Liu, Y. Li, H. Wang, X. Wan, Z. Wen, F. Fan, J. Zhang, K. Ariga, *Appl. Catal. B: Environ.* **2021**, *293*, 120213.
[29] J. Song, H.-Y. Su, G. Zhong, Y. Liu, X. Huang, J. Zhou, R. Miao, C. Li, W. Liao, X. Fu, S. Kang, Z.-Y. Lim, H. Liu, X. Li, A. You, F. Peng, *Colloid. Surfaces A* **2023**, *656*, 130417.
[30] S. Hu, X. Sun, Y. Zhao, W. Li, H. Wang, G. Wu, *J. Taiwan Inst. of Chemical E.* **2020**, *107*, 129-138.
[31] F. Xue, C. Zhang, H. Peng, L. Sun, X. Yan, F. Liu, W. Wu, M. Liu, L. Liu, Z. Hu, C.-W. Kao, T.-S. Chan, Y. Xu, X. Huang, *Nano Energ.* **2023**, *117*, 108902.
[32] Y. Fu, C. a. Liu, M. Zhang, C. Zhu, H. Li, H. Wang, Y. Song, H. Huang, Y. Liu, Z. Kang, *Adv. Energ. Mater.* **2018**, 8, 1802525.
[33] Z. Wu, X. Li, Y. Zhao, Y. Li, K. Wei, H. Shi, T. Zhang, H. Huang, Y. Liu, Z. Kang, *ACS Appl. Mater. Interfaces* **2021**, *13*, 60561–60570.
[34] D. Zeng, F. He, Y. Li, *Appl. Catal. A: Gen.* **2023**, *650*, 118986.
[35] Q. Ju, H. Huo, C. Huang, T. Wang, X. Liu, Z. Liang, L. Zhang, J. Ma, E. Kan, A. Li, *Chem. Eng. Sci.* **2024**, *284*, 119507.
[36] L. Zhang, W. Dong, Y. Zhang, X. Song, H. Jiang, *Chem. Eng. J.* **2023**, *472*, 144898.
[37] Z. Fan, H. Lu, Y. Liu, C. Chang, X. Guo, G. Long, Z. Jin, *Chem. Eng. J.* **2023**, *477*, 147008.
[38] C. Wang, C. Hu, F. Chen, H. Li, Y. Zhang, T. Ma, H. Huang, *Adv. Funct. Mater.* **2023**, *33*, 2301144.
[39] Z. Guo, Y. Tian, G. Dou, Y. Wang, J. He, H. Song, *Catal. Sci. Technol.* **2023**, *13*, 2714–2727.
[40] Y.-C. Huang, Y. Li, K. T. Arul, T. Ohigashi, T. T. T. Nga, Y.-R. Lu, C.-L. Chen, J.-L. Chen, S. Shen, W.-F. Pong, C.-L. Dong, W.-C. Chou, *ACS Sustainable Chem. Eng.* **2023**, *11*, 5390–5399.
[41] L. Sun, H. Peng, F. Xue, S. Liu, Z. Hu, H. Geng, X. Liu, D. Su, Y. Xu, X. Huang, *Sci. China Chem.* **2023**, *67*, 855–861.
[42] J. Liu, Y. Zhang, L. Lu, G. Wu, W. Chen, *Chem. Commun.* **2012**, *48*. 8826–8828.
[43] Y. Zhu, C. Lv, Z. Yin, J. Ren, X. Yang, C. L. Dong, H. Liu, R. Cai, Y. C. Huang, W. Theis, S. Shen, D. Yang, *Angew. Chem. Int. Ed.* **2019**, *59*, 868–873.
[44] F. Liang, X. Sun, S. Hu, H. Ma, F. Wang, G. Wu, *Diam. Relat. Mater.* **2020**, *108*, 107971.
[45] Y. Liu, Y. Zhao, Y. Sun, J. Cao, H. Wang, X. Wang, H. Huang, M. Shao, Y. Liu, Z. Kang, *Appl. Catal. B: Environ.* **2020**, *270*, 118875.
[46] N. Liu, S. Mohajernia, N. T. Nguyen, S. Hejazi, F. Plass, A. Kahnt, T. Yokosawa, A. Osvet, E. Spiecker, D. M. Guldi, P. Schmuki, *ChemSusChem* **2020**, *13*, 4937–4944.
[47] Y. Dong, Q. Han, Q. Hu, C. Xu, C. Dong, Y. Peng, Y. Ding, Y. Lan, *Appl. Catal. B: Environ.* **2021**, *293*, 120214.
[48] M. Zhu, C. Zhu, D. Wu, X. Wang, H. Wang, J. Gao, H. Huang, C. Shi, Y. Liu, Z. Kang, *Nanoscale* **2019**, *11*, 15984–15990.
[49] H. Jiang, W. Ye, H. Zhen, X. Luo, V. Fominski, L. Ye, P. Chen, *Chinese Chem. Lett.* **2025**, *36*, 109984.
[50] Z.-Z. Liang, X.-A. Li, Q.-Z. Chen, X.-L. Wang, P.-Y. Su, J.-F. Huang, Y. Zhou, L.-M. Xiao, J.-M. Liu, *ACS Catal.* **2024**, *14*, 10447–10461.
[51] Q. Zhang, S. Yuan, H. Yin, J. Yang, Z. Guan, *J. Mater. Chem.* **2024**, *12*, 18204–18213.
[52] M. Yu, J. Huang, Q. Niu, L. Li, Y. Yu, J. Bi, *Angew. Chem. Int. Ed.* **2025**, *64*, e202504533.
[53] Z. Lin, B. Wu, Y.-C. Huang, K. Zhang, Y. Wang, R. Wang, F. Zhang, J. Chen, S. Shen, *Artif. Photosynth.* **2026**, DOI: 10.1021/aps.5c00038.
